# Hormonal regulation and crosstalk during early endosperm and seed coat development

**DOI:** 10.1007/s00497-024-00516-8

**Published:** 2024-12-26

**Authors:** R. Pankaj, R. B. Lima, D. D. Figueiredo

**Affiliations:** Max Planck Institute of Molecular Plant Physiology, Potsdam Science Park, Am Mühlenberg 1, 14476 Potsdam, Germany

**Keywords:** Seed development, Phytohormone, Endosperm, Seed coat

## Abstract

**Key message:**

This review covers the latest developments on the regulation of early seed development by phytohormones.

**Abstract:**

The development of seeds in flowering plants starts with the fertilization of the maternal gametes by two paternal sperm cells. This leads to the formation of two products, embryo and endosperm, which are surrounded by a tissue of maternal sporophytic origin, called the seed coat. The development of each of these structures is under tight genetic control. Moreover, several phytohormones have been shown to modulate the development of all three seed compartments and have been implicated in the communication between them. This is particularly relevant, as embryo, endosperm, and seed coat have to coordinate their development for successful seed formation. Here, we review the latest advances on the hormonal regulation of early seed development in the model plant species *Arabidopsis thaliana*, with a focus on the endosperm and the seed coat. Moreover, we highlight how phytohormones serve as mechanisms of non-cell autonomous communication between these two compartments and how they are determinant in shaping seed formation.

## Introduction

The life cycle of vascular plants encompasses the alternation between diploid sporophytic and haploid gametophytic generations. The formation of a new sporophytic generation relies on the fertilization of the female gametophyte (or embryo sac) by paternal gametophytic sperm cells carried by the pollen tube. In angiosperms, each gametophyte provides two gametes: the pollen tube carries two sperm cells, supposedly functionally equivalent (Ingouff et al. 2009; Hamamura et al. 2011), while the female ovule contains two distinct gametes: the egg cell and the central cell (Drews and Koltunow 2011). Thus, a double fertilization event takes place, which leads to the formation of two products: the embryo, forming the next generation, and the endosperm, which nourishes the embryo (Bleckmann et al. 2014). These two structures are surrounded and protected by the seed coat, which derives from the maternal ovule integuments (Matilla 2019). These reproductive events are under tight genetic control and many of their aspects are regulated by phytohormonal pathways. The processes of ovule development and embryogenesis are strongly modulated by hormonal cues, but those topics were recently reviewed elsewhere (Barro-Trastoy et al. 2020, 2024; Verma et al. 2021). Similarly, the role of hormones in seed maturation, dormancy and germination has also been assessed in recent reviews (Shu et al. 2016; Tuan et al. 2018; Matilla 2020; Ali et al. 2022; Pan et al. 2023). Therefore, here we focus on how different plant hormones modulate the formation of the early endosperm and of the seed coat. We focus mainly on the state-of-the-art in the model species *Arabidopsis thaliana* (Arabidopsis), and provide examples of what is known in other species, where relevant or where information on Arabidopsis is lacking. In order to provide context for the readers, we provide a brief description of ovule development, as well as of early stages of seed formation.

## Stages of ovule development and gametogenesis

**Fig. 1:**
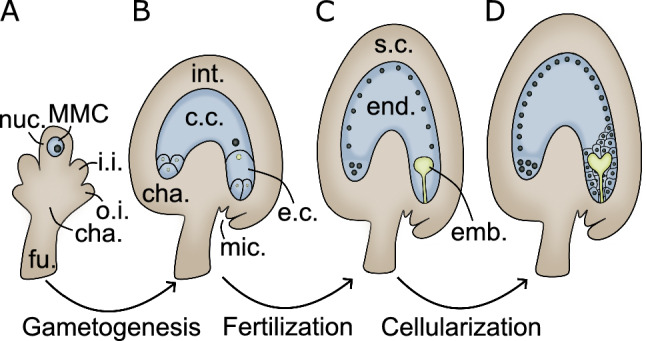
Stages of ovule and seed formation. **A** The gametophytic generation will originate from the megaspore mother cell (MMC), which differentiates embedded in nucellar tissue (nuc). The ovule integuments originate from the chalazal region (cha). In Arabidopsis there are two integuments, inner (i.i.) and outer (o.i.). The ovule is connected to the placenta by the funiculus (fu.). **B** The mature megagametophyte contains two gametes: egg cell (e.c.) and central cell (c.c.), which are surrounded by the integuments (int.). A micropylar opening (mic.) allows for the pollen tube to enter. **C** Upon fertilization two products form: embryo (emb.) and endosperm (end.), which are surrounded by the seed coat (s.c). **D** In most species the endosperm starts development as a coenocyte, which later cellularizes, except for the chalazal-most endosperm

In Arabidopsis, ovule primordia develop from finger-like projections on the placental tissue of the ovary (Schneitz et al. 1995). These projections can be divided into three main zones: the funiculus, which links the ovule to the placenta; the chalaza, an intermediate zone from which the integuments are formed; and the nucellus, which is the most distal from the placenta, and where the new gametophytic generation will arise (Fig. 1A). The process of sporogenesis and gametogenesis leading to the formation of female gametes varies slightly depending on the species. These variations are often related to the number of haploid spores that develop into a gametophyte and the number of mitotic divisions that lead to a mature gametophyte (Schmid et al. 2015). The most frequent kind of gametophyte is monosporic of the Polygonum type, which is characteristic of more than 70% of known plant species (Drews and Koltunow 2011; Schmid et al. 2015). Thus, here we focus on this type of megagametogenesis.

During the early stages of ovule development, a sub-epidermal cell in the nucellus gives rise to a megaspore mother cell (MMC), while the remaining sporophytic cells maintain their somatic fate (Drews and Koltunow 2011). The MMC can be easily identified by its larger size, and because it has a thicker cytoplasm and a larger nucleus than somatic cells. The MMC then enlarges and lengthens, undergoes meiosis, and generates four one-nucleate, haploid megaspores. Only one of the four megaspores survives in most cases. In Arabidopsis, it is typically the chalazal-most megaspore (Drews and Koltunow 2011). This surviving megaspore enters gametogenesis where it undergoes three consecutive rounds of mitosis, without cytokinesis, resulting in eight haploid nuclei. Two of those nuclei, called the polar nuclei, then move from each pole to the center of the developing female gametophyte, where they fuse. This step is followed by cellularization of the gametophyte, which produces a seven-celled structure composed of three antipodal cells, one central cell, two synergid cells, and one egg cell: forming the embryo sac (Fig. 1B) (Drews and Koltunow 2011). The central cell is homodiploid as it results from the fusion of two identical haploid nuclei, whereas the other cells receive just one haploid nucleus.

The female gametophyte is then progressively enclosed within a maternally-derived sporophytic tissue, called the integuments (Fig. 1B). These are formed from the chalazal region of the ovule primordium (Robinson-Beers et al. 1992; Bajon et al. 1999). The number of cell layers in the integuments varies depending on the species: for instance, the Arabidopsis ovule has two integuments: inner and outer (thus called bitegmic), with a total of five cell layers (Coen and Magnani 2018). These tissues begin forming as an epidermal layer that protrudes from the chalaza and surrounds the MMC. The MMC meiosis is accompanied by a periclinal division of the epidermal layer that surrounds it. This gives rise to two sporophytic layers: the inner and outer integument (Schneitz et al. 1995). The outer integument expands and elongates around the inner integument as the ovule develops, eventually enclosing the embryo sac. The inner integument develops more slowly than the outer integument and eventually joins with it at the micropyle, which is an opening that the pollen tube will grow through. Later, both integuments undergo additional periclinal divisions. In Arabidopsis, these divisions result in the production of five cell layers that surround the gametophyte: two from the outer integument and three from the inner integument (Robinson-Beers et al. 1992; Bajon et al. 1999). The asymmetric growth of the integuments leads to a characteristic curvature of the ovule, placing the micropyle in the vicinity of the funiculus (Vijayan et al. 2021). The mature ovule thus consists of two main structures: the megagametophyte and the surrounding sporophytic tissues (Fig. 1B).

## Double fertilization

The male gametophyte, or pollen grain, contains three cells: one vegetative nucleus and two gametes, the sperm cells. The pollen grains germinate once they land on a compatible stigma. There, the vegetative cell produces a tube that grows inside the pistil and towards the ovules while carrying the sperm cells. The pollen tube's passage within the pistil and arrival at the ovule relies on chemical signals and numerous interactions with sporophytic style tissues (Chae and Lord 2011; Guan et al. 2013; Baillie et al. 2024). The pollen tube enters the ovule via the micropyle and bursts as it reaches one of the synergid cells, where it discharges the sperm cells (Hamamura et al. 2011; Bleckmann et al. 2014; Lausser and Dresselhaus 2010). Those gametes then fertilize their maternal counterparts, the egg cell and central cell.

As previously mentioned, both male and female gametophytes contain two gametes each, and upon arrival of the pollen tube to the ovule, the two identical sperm cells fertilize the egg cell and the central cell. The resulting embryo and endosperm develop synchronously while wrapped in the maternal seed coat as a result of the double fertilization event (Fig. 1C) (Ingram 2010). The seeds remain connected to the mother via the funiculus, through which nutrients flow into the chalaza.

## The three components of the seed: embryo, endosperm, and seed coat

### Embryo: the next generation

One of the sperm cells undergoes karyogamy with the egg cell to generate the diploid zygote. Shortly after fertilization, there is a maternal-to-zygotic transcriptome transition, which is required for the kickstart of embryogenesis (Zhao et al. 2019; Kao and Nodine 2019). The zygote first divides asymmetrically, producing a small apical cell and a larger basal cell. The apical cell gives rise to the embryo proper, while the basal cell produces the suspensor, which connects the embryo to the maternal tissue. The apical cell divides numerous times in a very defined pattern to generate the proembryo which contains undifferentiated cells (ten Hove et al. 2015). The establishment of an apical-basal axis at the embryo is accompanied with radial patterning, a process that leads to the formation of the epidermal layer and of the ground and provascular tissues (Huang et al. 2023). The embryo begins to develop a heart shape at the next stage, with the cotyledons developing at the apical end and the root and hypocotyl precursors developing at the basal end (Palovaara et al. 2016). The cells that originate the shoot apical meristem become evident as well. In the following stage of development, the embryo elongates and takes on a torpedo-like shape. The cotyledons become more visible, and the root and hypocotyl precursors lengthen even more. Finally, the embryo matures, with well-defined cotyledons, a conspicuous shoot apical meristem, and a well-developed root (Palovaara et al. 2016). The rapid growth is accompanied by the buildup of storage substances including lipids and proteins, as well as the consumption of surrounding endosperm (Baud et al. 2008). At this point, the embryo maturation phase initiates and cell division and proliferation are replaced by cell expansion (Verma et al. 2022). At maturity, the Arabidopsis seed cavity is primarily made up of the bent dicotyledonar embryo, which goes into dormancy until the necessary germination circumstances are fulfilled (Baud et al. 2008).

### The nourishing endosperm

In most species, the fertilization of the diploid central cell with a haploid sperm cell originates a triploid endosperm. In the initial stages of development, the endosperm cavity is mainly occupied by the central vacuole that functions as the major sink of the seed (Brown et al. 1999; Olsen et al. 1999). Sucrose is transported from the maternal seed coat to the central vacuole, where it is converted into hexoses (Morley-Smith et al. 2008). While in the early stages of development sucrose reaches the embryo proper through the suspensor, in later stages the embryo is nourished directly from the endosperm (Morley-Smith et al. 2008; Lafon-Placette and Köhler 2014). Therefore, one of the main functions of the endosperm is to provide nutrients to the growing embryo. In addition, the endosperm also provides information to the surrounding tissues, in order to ensure coordinated seed development, as we will discuss below.

In Arabidopsis and cereals, as well as in most flowering plant species, the development of the endosperm initiates with free nuclear divisions without cytokinesis resulting in a multinucleate coenocyte (Olsen 2004). This developmental phase is referred to as the coenocytic endosperm (Fig. 1C). It is in the coenocytic phase that distinct domains are set within the endosperm: the micropylar domain can be distinguished by fusiform nuclei nearby the zygote, the peripheral domain by spherical nuclei evenly spaced within a thin peripheral layer, and the chalazal domain by large nuclei and dense cytoplasm (Brown et al. 2003; Olsen 2004). The coenocytic phase is followed by the cellularization phase, which is characterized by cell wall deposition and endosperm differentiation (Fig. 1D) (Li and Berger 2012; Köhler et al. 2021). In Arabidopsis, endosperm cellularization occurs as a wave, which initiates at the micropylar endosperm and then progresses to the peripheral endosperm, while the chalazal endosperm remains uncellularized (Olsen 2004; Ali et al. 2023). Therefore, when cellularization around the embryo is completed, the peripheral endosperm is still coenocytic. The cellularization of the endosperm leads to a decrease in the size of the central vacuole, which up to cellularization was the major reservoir of hexoses in the seed (Morley-Smith et al. 2008). From this developmental time point onwards, the transport of sucrose starts to occur directly from the endosperm to the embryo, shifting the major seed sink from the vacuole to the embryo (Lafon-Placette and Köhler 2014). Thus, the transition from a coenocytic to a cellularized endosperm is critical for embryo survival (Hehenberger et al. 2012; Köhler et al. 2021). This is demonstrated by the arrest of embryo development in mutants whose endosperm fails to cellularize and maintain the central vacuole as the major sucrose sink (Hehenberger et al. 2012). Furthermore, it was recently demonstrated that cellularization is necessary for the embryo to acquire tolerance to dehydration, which is crucial for embryo survival during seed maturation (Xu et al. 2023). After cellularization, the cell divisions cease, and reserves are accumulated. These processes integrate the maturation phase of nuclear endosperm development (Li and Berger 2012). In Arabidopsis, the endosperm undergoes programmed cell death and at maturity only one layer of peripheral endosperm is left surrounding the embryo. On the other hand, the endosperm of cereals persists until seed maturity, and it serves a nourishing function for the germinating seedling.

### The protective seed coat

The seed coat (or testa) is a tissue that develops entirely from the maternal sporophyte and can respond to fertilization by differentiating and growing, even though it is not fertilized itself (Fig. 1C-D). The seed coat serves numerous functions that aid in the protection of the developing embryo and endosperm (Haughn and Chaudhury 2005; Radchuk and Borisjuk 2014). Following fertilization, the seed coat grows and makes room for the growing embryo and endosperm, and this is done by cell elongation rather than cell division (Garcia et al. 2003). As the seed coat develops, each layer undergoes distinct differentiation events. The accumulation of proanthocyanidins (PAs) begins soon after fertilization in the innermost layer, the endothelium (Debeaujon et al. 2003). PAs are necessary for seed dormancy, germination after long-term storage, and pathogen resistance (Debeaujon et al. 2000). Additionally, they are responsible for giving Arabidopsis and other Brassicaceae seeds their characteristic brown hue, which appears after the oxidation at the later stage of seed development (Shirley 1998; Debeaujon et al. 2003). Fertilization also causes the buildup of starch granules in the outermost layers of the seed coat, and as seed development advances, the outermost cell layer secretes mucilage into the area between the plasma membrane and the outer cell wall (Windsor et al. 2000). This causes cytoplasm to become constrained within the center of the cell, generating a columella structure. The starch that has accumulated is converted into secondary cell walls, which reinforces the columella (Windsor et al. 2000). The chalaza is a specialized seed coat region that has a direct connection with the funiculus and the maternal plant (Beeckman et al. 2000). This region contains xylem and phloem vessels that connect to the funiculus, allowing water and nutrients to be taken in (Khan et al. 2014; Millar et al. 2015). The chalazal seed coat serves as a loading zone for maternally produced nutrients, which are then transmitted to the remaining seed coat cell layers, as well as to the endosperm and embryo. These structures are present in the mature ovule and form in parallel with female gametogenesis; their identity is defined prior to fertilization and is solely controlled by the mother (Schneitz et al. 1995).

Later phases of seed development show a gradual collapse of the multiple seed coat cell layers: the two outermost layers of the inner seed coat are the first to collapse, followed by the innermost layer of the outer seed coat (Beeckman et al. 2000). Thus, the final mature seed coat is made up of two cell layers: the mucilage-rich outermost layer of the outer seed coat and the innermost layer of the inner seed coat. Between these two layers, an amorphous layer of collapsed cells accumulates the synthesized PAs (Beeckman et al. 2000; Windsor et al. 2000). PAs and mucilage are hypothesized to work together to separate the seed from the outer environment, allowing germination to occur only when certain conditions are satisfied (Haughn and Chaudhury 2005). When a seed comes into contact with water and begins to germinate, the mucilage in the seed coat rapidly releases, creating a humid environment that aids germination (Windsor et al. 2000; Haughn and Chaudhury 2005).

In the next sections we detail how endosperm and seed coat development is shaped by various hormones. In particular, auxin, gibberellin, brassinosteroids and cytokinin have been shown to play prominent roles in the development of these tissues. For each hormone we thus provide a short description of their biosynthesis and signaling pathways, in order to guide the reader. In each accompanying figure we include a simplified scheme of these pathways, as well as a diagram of the main developmental processes that each hormone is involved in during seed development.

## Auxin: a driver of endosperm and seed coat development

### Auxin biosynthesis and signaling

**Fig. 2:**
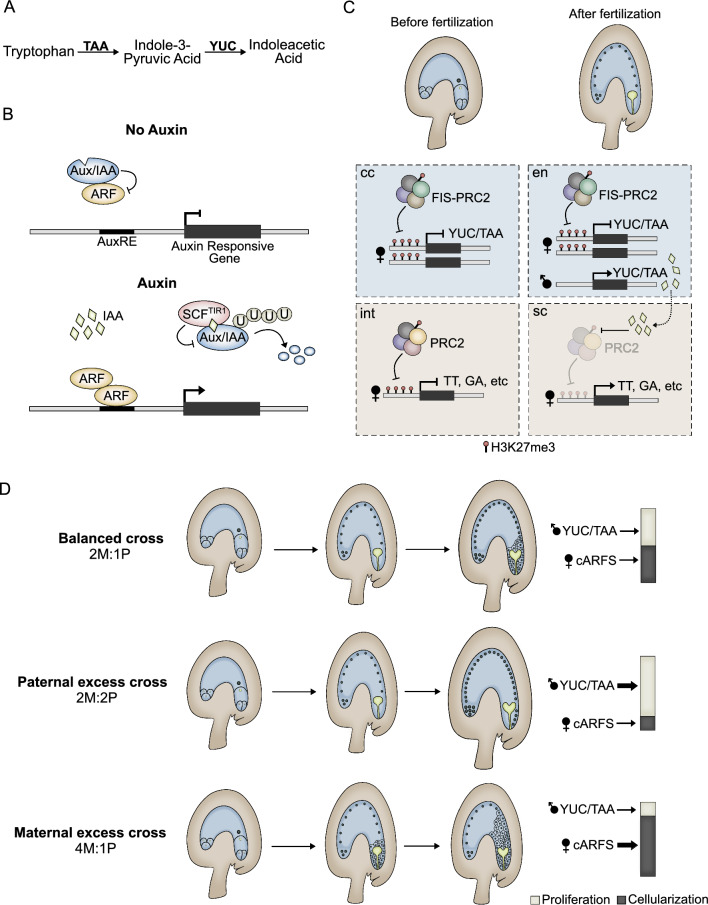
Auxin regulates endosperm and seed coat formation. **A** Two-step auxin biosynthesis pathway. **B** Auxin signalling relies on the degradation of Aux/IAA repressors by the SCF^TIR1^ complex, which allows for regulation of gene expression by ARFs. **C** Before fertilization, FIS-PRC2 blocks the expression of auxin biosynthesis genes in the megagametophyte and sporophytic PRC2s block the expression of seed coat genes. Paternal expression of auxin biosynthesis genes after fertilization leads to endosperm formation. Auxin is also proposed to be exported to the integuments, where it removes PRC2 function and allows for seed coat formation. TT, Transparent Testa pathway, GA, gibberellin related genes. **D** A balance between auxin biosynthesis and signalling also determines the time of endosperm cellularization. In paternal-excess crosses, increased auxin production and reduced pcARF activity lead to a delay of cellularization, while the opposite is true in maternal-excess crosses

Auxins are naturally occurring low-molecular weight molecules that induce growth (Sauer et al. 2013). The most abundant and well-characterized auxin is indoleacetic acid (IAA), and its biosynthesis can occur dependently or independently of tryptophan (Trp), the former route being the better characterized (Mano and Nemoto 2012). This route can be divided into four different pathways that differ from each other based on whether the Trp derivative used as substrate is indole-3-acetaldoxime (IAOx), indole-3-acetamide (IAM), indole-3-pyruvic acid (IPA), or tryptamine (TAM) (Gomes and Scortecci 2021). Given that the IPA pathway is considered the predominant auxin biosynthesis pathway in Arabidopsis (Mashiguchi et al. 2011), we focus exclusively on this route. The biosynthesis of IAA via IPA consists of a two-step pathway: first, Trp is converted into IPA by the TRYPTOPHAN AMINOTRANSFERASE OF ARABIDOPSIS (TAA) family of aminotransferases and then, the YUCCA (YUC)-type flavin-containing monooxygenases convert IPA to IAA (Fig. 2A) (Zhao et al. 2001; Stepanova et al. 2008; Mashiguchi et al. 2011; Won et al. 2011). Even though this biosynthetic pathway is straightforward, multiple homologous genes encode the enzymes required for IAA biosynthesis, meaning that local auxin production relies on spatial and temporal expression of certain *TAA* and *YUC* combinations (Cheng et al. 2006, 2007; Zhao 2018).

Perception of auxin then occurs in the nucleus, when the hormone binds to the receptor TRANSPORT INHIBITOR RESISTANT 1/AUXIN SIGNALING F-BOX (TIR1/AFB), promoting its interaction with the transcriptional repressors AUXIN/INDOLE-3-ACETIC ACIDs (AUX/IAAs) (Lavy and Estelle 2016). Thereby, auxin binding results in SCF^TIR/AFB^-mediated ubiquitination, and consequent degradation of AUX/IAAs. In the absence of auxin, the AUX/IAAs inhibit the TFs ARABIDOPSIS RESPONSE FACTORS (ARFs), which modulate auxin responses by binding to *cis*-regulatory sequences referred to as auxin response elements (AuxREs) (Ulmasov et al. 1999; Tiwari et al. 2003). Therefore, the auxin-induced degradation of the AUX/IAA releases ARFs from repression enabling auxin responses (Fig. 2B) (Gray et al. 2001; Dos Santos Maraschin et al. 2009).

### Regulation of endosperm development by auxin

Auxins have long been known to be involved in seed formation, as these hormones are often found in endosperms of several species (Avery et al. 1942; Teubner 1953; Jennings 1971; Lur and Setter 1993; Abu-Zaitoon et al. 2012). In fact, auxin was demonstrated to be a post fertilization trigger for central cell replication, and thus endosperm formation (Figueiredo et al. 2015). This is supported by observations that ectopic expression of auxin biosynthesis genes in the unfertilized central cell, or the exogenous auxin application to unpollinated pistils, results in endosperm development without fertilization (Figueiredo et al. 2015). The initiation of auxin biosynthesis only after fertilization agrees with the notion that developmental pathways leading to endosperm formation are actively suppressed before gamete fusion (Makarevich et al. 2006). Furthermore, sperm cell entry in the central cell is sufficient to induce a few rounds of central cell replication, suggesting that the sperm cell carries factors that might lift that suppression (Aw et al. 2010). Indeed, de novo auxin biosynthesis after fertilization could be attributed to the parental biased expression of the Arabidopsis *YUC10* and *TAR1* alleles, which are repressed in the central cell, but not in the male gametes (Gehring et al. 2011; Hsieh et al. 2011; Wolff et al. 2011; Figueiredo et al. 2015). This phenomenon of parent-of-origin differential gene expression, referred to as genomic imprinting, results from the distinct activity of epigenetic pathways in the maternal and paternal germlines, leading to the same DNA sequence carrying different epigenetic marks (Batista and Köhler 2020). The silencing of many maternal alleles requires the activity of the POLYCOMB REPRESSIVE COMPLEX 2 (PRC2) (Batista and Köhler 2020). The PRC2 is a multi-subunit complex of Polycomb Group proteins (PcG) that modifies chromatin structure by depositing trimethylation marks on lysine 27 of histone 3 (H3K27me3) (Godwin and Farrona 2022; Vijayanathan et al. 2022). In Arabidopsis ovules, three PRC2 complexes can be distinguished: the FERTILIZATION INDEPENDENT (FIS) complex in the central cell, composed by the subunits FIS2, MEDEA (MEA), FERTILIZATION INDEPENDENT ENDOSPERM (FIE), and MULTICOPY SUPPRESSOR OF IRA 1 (MSI1); and the EMBRYONIC FLOWER 2 (EMF2) and VERNALISATION 2 (VRN2) complexes in the integuments, composed by either EMF2 or VRN2 plus the subunits FIE, MSI1, and CURLY LEAF (CLF) or SWINGER (SWN) (Roszak and Köhler 2011; Mozgova et al. 2015). Therefore, according to their expression and function on the plant tissues, the FIS complex can be described as the gametophytic PRC2 (Fig. 2C, “cc” panel), and the EMF and VRN complexes as the sporophytic PRC2s (Fig. 2C, “int” panel), respectively. The interplay between DNA and histone methylation in maternal and paternal alleles underlies the different mechanisms that give rise to genomic imprinting (Moreno-Romero et al. 2019; Batista and Köhler 2020). In the central cell, which is hypomethylated, the gametophytic PRC2 silences the maternal alleles encoding YUC10 and TAR1 via H3K27me3 deposition (Fig. 2C, “cc” panel). Upon fertilization, the active *YUC10* and *TAR1* paternal alleles are introduced in the central cell (Fig. 2C, “en” panel). Given that PRC2 recruitment to its target loci is seemingly prevented by DNA methylation (Weinhofer et al. 2010), the paternally-derived *YUC10* and *TAR1* are protected from PRC2 activity and their expression leads to auxin biosynthesis (Fig. 2C, “en” panel) (Figueiredo et al. 2015).

The type I MADS-box TF AGAMOUS-LIKE 62 (AGL62) was also proposed to be required for auxin biosynthesis in the endosperm, both in Arabidopsis and in strawberry (Guo et al. 2022). However, FveAGL62 does not seem to directly regulate the expression of auxin biosynthesis genes in the endosperm, but does it indirectly via the regulation of genes encoding ATHB TFs (Guo et al. 2022). Importantly, another MADS-box TF, PHERES1 (PHE1), has been shown to target *YUC10* (Batista et al. 2019b). These observations suggest that the post-fertilization expression of auxin biosynthesis genes is under tight regulation, both by PRC2 and by the direct and indirect action of MADS-box TFs.

Although post-fertilization produced auxin triggers central cell replication leading to the formation of the endosperm, the mechanism by which auxin stimulates central cell replication remains elusive. Linking the FIS-PRC2 repression of auxin biosynthesis to endosperm initiation, is the observation that mutants for subunits of FIS-PRC2, such as *fis2* and *mea*, can initiate endosperm development without fertilization (Ohad et al. 1996; Chaudhury et al. 1997; Grossniklaus et al. 1998; Makarevich et al. 2006). This coincides with the expression of *YUC10* in those maternally derived asexual endosperms and, concomitantly, with ectopic auxin activity (Figueiredo et al. 2015). Taken together, these observations demonstrated that central cell replication is blocked prior to fertilization through the gametophytic PRC2-mediated silencing of auxin biosynthesis genes. Interestingly, the genomic imprinting of auxin biosynthesis in the endosperm seems to be a feature of angiosperms, as it has been demonstrated in both eudicots and monocots (Luo et al. 2011; Waters et al. 2013; Xin et al. 2013; Du et al. 2014; Hatorangan et al. 2016; Klosinska et al. 2016). In fact, the paternal expression of auxin biosynthesis genes in the seed nourishing tissues can even be traced back to early diverging angiosperms, suggesting that the mechanisms of seed initiation may be conserved in all angiosperms (Florez-Rueda et al. 2023, 2024). Consistent with this, mutations in auxin biosynthesis genes have been shown to lead to endosperm defects in several species, including rice and maize (LeClere et al. 2008; Bernardi et al. 2012; Figueiredo et al. 2015; Xu et al. 2021). Moreover, PRC2 mutants also lead to asexual endosperm formation in rice (Tonosaki et al. 2021), and these mutations also correlate with the ectopic expression of auxin biosynthesis paternally-expressed genes (Wu et al. 2023). This includes *OsYUC11*, which is necessary for proper endosperm formation in rice (Xu et al. 2021). Interestingly, mutations in its homologue in maize, *ZmYUC1*, also coincides with endosperm defects (Bernardi et al. 2012).

In addition to its role in triggering and promoting early endosperm proliferation, auxin also regulates endosperm cellularization (Fig. 2D). The inheritance of two copies of the maternal genome (2M) and one copy of the paternal (1P) in the endosperm is crucial for successful seed formation (Lafon-Placette and Köhler 2016). In crosses where these ratios are disturbed, the timing of endosperm cellularization is altered: while in maternal excess crosses (4n × 2n, 4M:1P) cellularization occurs earlier than in balanced crosses (2n × 2n, 2M:1P), in paternal excess crosses (2n × 4n, 2M:2P) the cellularization process is delayed or does not take place at all (Fig. 2D) (Scott et al. 1998). Given that the outcome of crosses with either maternal or paternal excess is an aborted seed with a triploid (3n) embryo, this phenomenon is referred to as triploid block. Curiously, the triploid seeds of paternal-excess crosses show increased expression of genes involved in auxin biosynthesis, signaling, and transport, in comparison to seeds from balanced crosses (Batista et al. 2019a). Furthermore, overexpression of auxin biosynthesis genes in the endosperm results in failed cellularization, similar to what is observed in paternal-excess crosses (Batista et al. 2019a). These observations suggested that increased auxin biosynthesis is the underlying cause of endosperm cellularization failure in triploid seeds. This was further supported by evidence demonstrating that in paternal-excess crosses, endosperm cellularization could be restored by reducing auxin signaling (Batista et al. 2019a). In fact, the expression of genes involved in auxin biosynthesis and signaling was shown to decrease during endosperm cellularization in 2n seeds, but to remain upregulated in 3n seeds (Batista et al. 2019a). Among the genes identified as upregulated were a cluster encoding redundant pericentromeric ARFs (pcARFs), which were preferentially or exclusively expressed from the maternal alleles (Batista et al. 2019a; Butel et al. 2024). Analysis of ARF22 expression during seed development revealed that this auxin response factor was detected in the endosperm at the onset of cellularization, and that in maternal and paternal excess crosses, its expression was precocious or delayed, respectively, just like endosperm cellularization (Butel et al. 2024). This indicated that the accumulation of pcARFs coincides with the initiation of the cellularization process, which was further demonstrated by delayed cellularization in high order *pcARF* mutant seeds (Butel et al. 2024). In agreement with pcARFs promoting endosperm cellularization, expression of ARF22 in early endosperm development led to early cellularization (Butel et al. 2024). Taken together, these studies show that auxin homeostasis must be kept in order to maintain timely expression of these repressive pcARFs and ensure proper cellularization timing. It is interesting to note that delayed endosperm cellularization resulting from paternal excess crosses leads to increased auxin signaling (Batista et al. 2019a), but to delayed expression of pcARFs (Butel et al. 2024). This may point towards a maternal and paternal antagonistic control of endosperm cellularization on auxin biosynthesis and signaling (Fig. 2D).

### Auxin as a seed coat initiation signal

The initiation of seed coat development is dependent on the formation of the endosperm: if the central cell is not fertilized, a seed coat will not develop, even if an embryo is formed (Weijers et al. 2003; Aw et al. 2010; Roszak and Köhler 2011). In addition, integument differentiation occurs in mutants that can initiate central cell replication without fertilization (Ingouff et al. 2006). Therefore, an endosperm-derived signal was hypothesized to be required for seed coat development (Roszak and Köhler 2011). Figueiredo et al. (2016) revealed that the auxin produced in the endosperm after fertilization drives seed coat development (Fig. 2C, bottom panels). This was supported by previous observations that auxin reporters were activated in seeds shortly after fertilization (Dorcey et al. 2009). Similar to the role of FIS-PRC2 in repressing the maternal genes encoding auxin biosynthesis enzymes, in the integuments, the sporophytic PRC2s block genes that are necessary for the development of the seed coat (Fig. 2C, “int” panel) (Roszak and Köhler 2011; Figueiredo et al. 2016). This PRC2-mediated repression is lifted by the auxin produced in the central cell upon fertilization, which is transported to the integuments (Fig. 2C, right panels) (Figueiredo et al. 2016). Such a seed coat initiation signal had been proposed to be under the control of the type I MADS-box TF AGL62, which is expressed during early endosperm development and is a regulator of endosperm proliferation and cellularization (Kang et al. 2008; Hehenberger et al. 2012; Figueiredo et al. 2015, 2016). And indeed, although AGL62 is only expressed in the endosperm, *agl62* seeds fail to develop a seed coat, a phenotype that was attributed to auxin depletion from the integuments (Roszak and Köhler 2011; Figueiredo et al. 2016). Strikingly, while auxin was absent from the integuments, it accumulated in the endosperm, indicating that in *agl62* seeds the export of auxin from the endosperm to the integuments is impaired (Figueiredo et al. 2016). These observations further supported the hypothesis that auxin derived from the endosperm is required for seed coat formation. It was proposed that AGL62 could possibly mediate the export of auxin from the endosperm to the integuments by regulating the expression of the gene encoding the ABCB-type transporter PGP10 (Figueiredo et al. 2016). Interestingly, a recent work described rather a depletion of auxin activity in *agl62* endosperms (Guo et al. 2022). The reason for these conflicting results is still unclear, and could be attributed to the use of different auxin sensors (Guo et al. 2022).

From the fertilization process to seed maturation, the formation of the seed coat comprises various stages of development. One of these stages is the thickening and differentiation of the endothelium, the seed coat’s inner layer, which confers mechanical strength to this tissue. The observation that *arf2* mutant seeds are larger than the ones from wildtype (WT) linked auxin signaling to this process (Schruff et al. 2006). The endothelium, as well as the outer integument layers, of *arf2* seed coats have more cells comparatively to the WT, which is caused by additional anticlinal cell divisions (Schruff et al. 2006). Therefore, auxin signaling is required in the maternal sporophytic tissues to control cell division. It was proposed that ARF2 could function in the same network as SEEDSTICK (STK) and GORDITA (GOA), two TFs that are expressed in the integuments and are important for seed development (Paolo et al. 2021).

Finally, recent evidence suggests that throughout development the seed coat also uptakes auxin from the maternal plant. The analysis of auxin distribution during seed development revealed high auxin activity in the chalazal seed coat and funiculus, where the auxin transporter PIN-LIKE 3 (PIN3) is also expressed (Liu et al. 2023). Moreover, the auxin biosynthesis genes *ANTHRANILATE SYNTHASE BETA SUBUNIT 1* (*ASB1)*, *TAA1*, *YUC1*, and *YUC6* were expressed in the funiculus suggesting local auxin biosynthesis in this tissue (Larsson et al. 2017; Robert et al. 2018; Liu et al. 2023). Thus, it was proposed that auxin produced in the funiculus is transported to the chalazal seed coat and from this seed region to the integuments. In agreement with this hypothesis, while auxin was almost undetectable in the seed coat of *pin3* mutants, it accumulated in the funiculus (Liu et al. 2023). Overall, these findings indicate that the funiculus likely serves as an additional source of auxin to the seed coat.

## Brassinosteroids dictate seed shape and endosperm proliferation

### BR biosynthesis and signaling

**Fig. 3:**
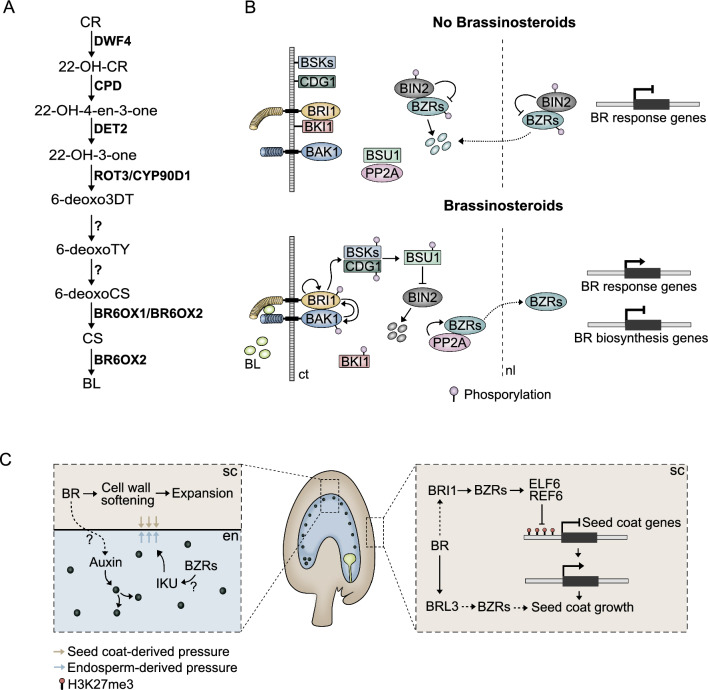
BRs regulate endosperm and seed coat formation. **A** Simplified linear BR biosynthesis pathway. **B** BR signalling relies on the recognition of BL by plasma membrane receptors, which trigger a phosphorylation cascade, culminating in the degradation of the negative regulator BIN2, and the release of the BZR/BES TFs into the nucleus, where they regulate the expression of BR-related genes. ct, cytosol; nl, nucleus. **C** Production of BR in the seed coat creates a permissive environment for cell wall expansion and seed coat growth, and these physical cues determine the division rates of the endosperm nuclei. The IKU pathway, which has been shown to regulate endosperm growth, is also proposed to be regulated by BR effector TF. Moreover, BR acts in concert with JMJ histone demethylases in the seed coat, and contributes to the removal of H3K27me3 marks, allowing for seed coat genes to be expressed and for the tissue to grow

Brassinosteroids (BRs) are plant steroid hormones that play an important role in plant development, growth and reproduction by eliciting a diverse set of physiological and morphological responses (Nolan et al. 2020; Lima and Figueiredo 2024). Among the 70 BRs identified until date, brassinolide (BL) is the most bioactive compound, followed by its precursor castasterone (CS) (Clouse 2011; Hayat et al. 2019). BR biosynthesis is a complex system, where BR precursors and products are involved in multiple metabolic grids that comprise several enzymes. Even so, the biosynthesis of BL can be simplified in a linear pathway, as represented in Fig. 3A. The enzymes known to be involved in the pathway are, in order from the hydroxylation of campesterol (CR) to the oxidation of CS into BL, DWARF4 (DWF4), CONSTITUTIVE PHOTOMORPHOGENIC DWARF (CPD), DE-ETIOLATED2 (DET2), CYP90C1/ ROTUNDIFOLIA3 (ROT3), CYP90D1, and BR6OX1 and OX2 (Chory et al. 1991; Li et al. 1996; Kim et al. 1998, 2005a, 2005b; Choe et al. 1998; Noguchi et al. 1999, 2000; Nomura et al. 2005; Kwon et al. 2005; Ohnishi et al. 2006, 2012). BRs are produced in the endoplasmic reticulum and transported to the apoplast where they are perceived (Ying et al. 2024).

The BR signaling cascade initiates when BL binds to the leucine-rich repeat receptor kinase BRASSINOSTEROID INSENSITIVE 1 (BRI1) and its homologs BRI-LIKE1 (BRL1) and BRL3 (Fig. 3B) (Li and Chory 1997; Caño-Delgado et al. 2004). The activation of BRI1 and its co-receptor EMBRYOGENESIS RECEPTOR KINASE 3/ BRI1-ASSOCIATED KINASE 1 (SERK3/BAK1), leads to the activation of the main BR response TFs BRASSINAZOLE RESISTANT1 (BZR1), BRI1-EMS-SUPPRESSOR1 (BES1), and their homologs BEH1-4 (Wang et al. 2002; Yin et al. 2002; Li et al. 2002; Kim et al. 2023). When BL is not present, these TFs are phosphorylated by the GSK3-like kinase BRASSINOSTEROID INSENSITIVE 2 (BIN2) and targeted for proteasomal degradation (Fig. 3B) (Yin et al. 2002; He et al. 2002). The binding of BL results in the activation of CONSTITUTIVE DIFFERENTIAL GROWTH1 (CDG1) and BR SIGNALING KINASES (BSKs) by BRI1, which in turn phosphorylate and activate BRI1 SUPPRESSOR 1 (BSU1) (Li and Nam 2002; Tang et al. 2008; Kim et al. 2011; Ren et al. 2019). The active BSU1 dephosphorylates BIN2, leading up to its degradation, freeing BZR1 and BES1 from BIN2-mediated repression. Upon being dephosphorylated by PROTEIN PHOSPHATASE 2A (PP2A), the BZRs accumulate in the nucleus and modulate the expression of BR responsive genes (Fig. 3B) (Sun et al. 2010; Yu et al. 2011; Tang et al. 2011).

### Regulation of endosperm development by BR

BRs are found not only in all plant vegetative organs, namely roots, stems and leaves, but also in all reproductive structures including anthers, gynoecia, ovules, pollen grains and seeds suggesting a role for this hormone in many aspects of plant reproduction (Lima and Figueiredo 2024). The link between BR and seed development arose from physiological studies reporting altered seed size, when BR levels were perturbed either by loss of genes involved in BR synthesis and/or signaling or by their overexpression (Choe et al. 2000; Kim et al. 2005b; Zhang et al. 2016, 2018). Notwithstanding, the molecular mechanisms that shape BR function throughout seed development and that distinguish the hormone’s role within the different seed compartments only started to emerge recently.

The role of BRs in regulating endosperm development was first proposed after the observation that in siliques of the BR biosynthesis mutant *det2*, the expression levels of genes integrating the endosperm-specific HAIKU (IKU) pathway were slightly reduced comparatively to the WT at 4–5 days after pollination (DAP) (Jiang et al. 2013). These genes included *IKU1*, *IKU2* and *MINISEED3* (*MINI3*), which together, regulate seed cavity expansion and coenocytic endosperm growth (Luo et al. 2005). Given that *det2* produces seeds that are smaller than the ones from WT, and that seed size could be rescued by exogenous BL application, this suggested that BRs could regulate seed size by modulating endosperm growth (Jiang et al. 2013). Indeed, BRs were demonstrated to promote early endosperm development as mutants with impaired BR biosynthesis and signaling show reduced endosperm proliferation in comparison to the respective WT (Lima et al. 2024). Curiously, reduced endosperm proliferation was also observed in the seeds of *bes1-D* and *bzr1-D* mutants, in which BR signaling is constitutively active, hinting for a dose-dependent effect of BRs in endosperm development (Lima et al. 2024). In agreement with this hypothesis, exogenous application of increasing BL concentrations together with auxin (the trigger of central cell replication) to unpollinated pistils, resulted in increased frequency of ovules producing fertilization-independent endosperm (Lima et al. 2024). This indicates that, similar to the hormone’s effect in other plant tissues, BRs promote endosperm growth in a dose-dependent manner (Vogler et al. 2014; Vukašinović et al. 2021; Lima et al. 2024). Surprisingly, during early endosperm development, the components of the BR signaling and biosynthesis pathways are only detected in the sporophytic seed tissues, but absent from the endosperm and the embryo, revealing that the effect of BRs in endosperm proliferation is of maternal origin (Fig. 3C) (Lima et al. 2024). Altogether, these observations indicate that while endosperm proliferation is regulated by BR signaling in sporophytic tissues, the zygotic products might contribute to that regulation in later stages of seed development. In fact, the number of endosperm nuclei and seed size of *iku2-3* and *mini3-1* seeds remains indistinguishable from the WT until 72 h post pollination (3 DAP), a time point from which the *iku2-3* and *mini3-1* endosperms initiate cellularization (Luo et al. 2005). Furthermore, it is noteworthy that the effect of both mutations in seed size are of zygotic nature (Luo et al. 2005). In fact, the small seed phenotype of *iku2* seeds was recently attributed to the stiffening of the outer cell wall of the middle seed coat layer (which has mechanosensitive properties), due to increased endosperm turgor pressure (Creff et al. 2023). Given that a time course determining the expression pattern of the BR signaling and biosynthesis genes throughout seed development is still lacking, it could be possible that there is a crosstalk between BRs and the IKU pathway later in development.

Since the seed size of BR mutants is smaller than the WT, it was hypothesized that the less proliferative endosperm observed in these mutants could be caused by physical constraints exerted by the seed coat. The reduced accumulation of methyl esterified pectins in the cell walls of *det2-1* and *bri1-6* seed coats, further supported that the maternal sporophytic tissues of these mutants are stiffer than in the WT (Lima et al. 2024). Coinciding with this, there is reduced auxin signaling in the endosperms of BR mutants, providing a possible explanation for the decreased nuclear replication rates (Fig. 3B) (Lima et al. 2024). In fact, genes encoding components of auxin signalling and transport are deregulated in BR mutants (Lima et al. 2024). This includes *PIN3*, which is expressed in the innermost seed coat layer surrounding the endosperm (Liu et al. 2023). Thus, it is possible that auxin transport from the seed coat to the endosperm could be deregulated in these BR mutants. Nevertheless, how the lack of BRs in the seed coat regulates auxin activity in the endosperm, remains to be explained (Fig. 3C).

### Regulation of seed coat development by BR

Several studies have also implicated BRs in the regulation of seed shape in several species. BR-deficient and insensitive mutants of Arabidopsis, *Oryza sativa*, *Pisum sativum*, and *Vicia faba* have abnormally shaped seeds when compared to the WT (Jiang and Lin 2013). For example, the rice BR deficient mutant *brd2* has shorter and smaller grains (Hong et al. 2005), while the *dwf11* mutant has smaller seeds (Tanabe et al. 2005). This gene, *DWF11*, encodes a rice cytochrome P450, which is involved in BR biosynthesis. Furthermore, the rice dwarf mutant *d61* produces small seeds, which is due to the loss of function of a rice BR receptor, encoded by *OsBRI1* (Morinaka et al. 2006)*.* BR deficient *Vicia faba* mutants also make smaller seeds (Fukuta et al. 2006), as does the dwarf mutant *lk*, a severe BR deficient mutant in pea (Nomura et al. 2007). Overexpression of a BR-biosynthetic gene, on the other hand, has been demonstrated to boost rice seed filling and yield (Wu et al. 2008). In Arabidopsis, the BR-deficient mutant *dwf5* produces small seeds (Choe et al. 2000), whereas overexpression of the P450 monooxygenase family gene CYP72C1 in the dwarf mutant *shk1-D* reduces endogenous BR levels, resulting in general short organs and small seeds (Takahashi et al. 2005). The mature dry seeds of the BR-deficient mutant *det2* and the BR-insensitive mutant *bri1-5* (a weak allele of the *bri1* mutant) were discovered to be smaller than the equivalent WT seeds (Jiang et al. 2013). Furthermore, exogenous BR was shown to partially rescue *det2* seed size and weight, indicating BRs' positive regulatory role in seed size/mass (Jiang et al. 2013).

In Arabidopsis, the effect of BRs on seed shape and growth seems to be of maternal origin, at least in early stages of seed development (Pankaj et al. 2023). However, zygotic effects cannot be ruled out, as we described in the previous section (Jiang et al. 2013). The regulation of seed growth by BR was recently linked to their role in H3K27me3 homeostasis (Fig. 3C). Before fertilization, the development of the seed coat is blocked by sporophytically acting PRC2s (Roszak and Köhler 2011; Figueiredo et al. 2016). This means that H3K27me3 blocks the expression of seed coat fate genes (Fig. 3C). And although these complexes are removed by endosperm-derived auxin, as described above, this removal does not explain how seed coat genes become derepressed. Because the seed coat cells do not divide, but rather elongate, this means that dilution of the repressive marks is unlikely. Therefore, enzymatic removal of H3K27me is the most likely scenario. This removal is often catalyzed by JUMONJI (JMJ) histone demethylases (Yan et al. 2018). And, importantly, JMJ proteins have been shown to interact with the BR effector TFs BZR1 and BES1. While BES1 recruits the JMJs EARLY FLOWERING 6 (ELF6) and RELATIVE OF EARLY FLOWERING (REF6) to target loci (Yu et al. 2008), BZR1 was shown to interact with ELF6 but not with REF6 (Li et al. 2018). Consistent with this, *jmj* mutants have seed coat initiation phenotypes, and BR mutants are hypermethylated in H3K27me3 (Pankaj et al. 2023). Thus, seed coat initiation seems to rely on a concerted action of auxin and BRs, where the former ensures PRC2 removal from the integuments (Figueiredo et al. 2016), and the latter facilitates the removal of the H3K27me3 marks (Fig. 3C) (Pankaj et al. 2023). This concerted action leads to the expression of genes necessary for seed coat development, likely including those involved in the biosynthesis of another hormone, gibberellin (GA), which we discuss in the next section.

## Gibberellins as signals for seed coat formation

### GA biosynthesis and signalling

**Fig. 4:**
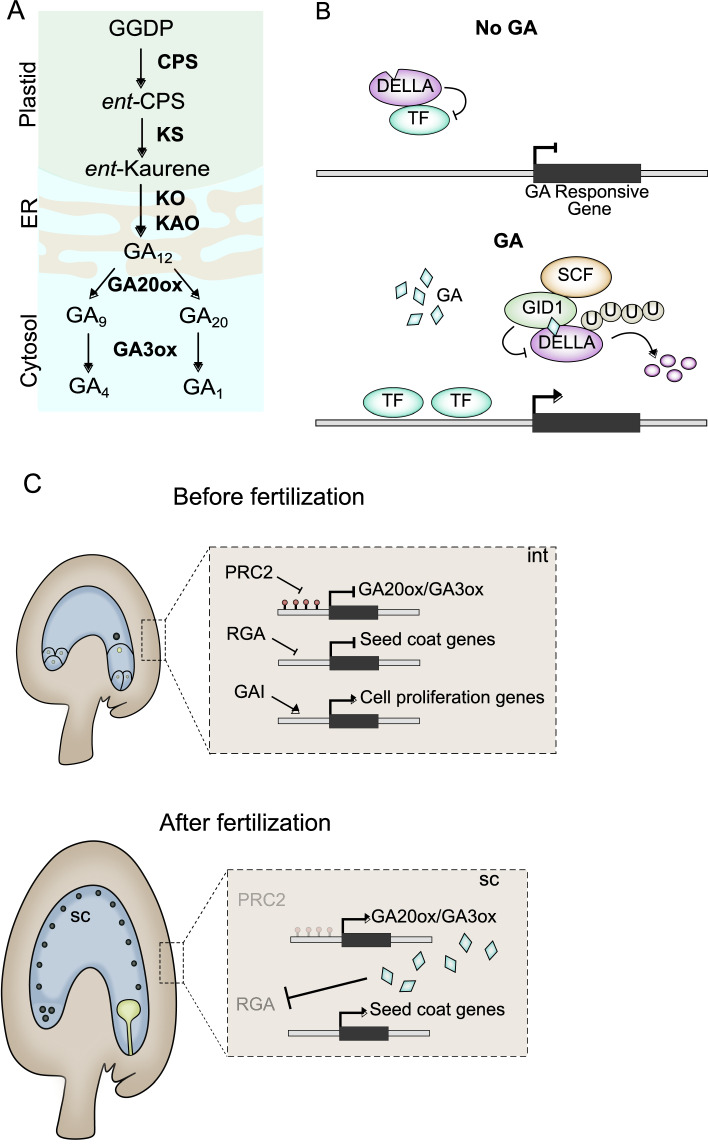
GAs regulate the development of the sporophytic tissues. **A** GA biosynthesis pathway, indicating the intracellular location of each step. **B** GA signalling relies on the GA-mediated degradation of DELLA proteins, which allows TFs to regulate GA-response genes. **C** Before fertilization the expression of GA biosynthesis genes is repressed by sporophytic PRC2s. DELLAs repress seed coat development genes and promote the proliferation of integument cells. **D** After fertilization the PRC2 repression is removed, which coincides with the expression of GA biosynthesis genes, likely leading to GA production in the integuments. This in turn leads to RGA removal, allowing for active GA signalling, which drives seed coat formation

Gibberellins (GAs) play pivotal roles in various aspects of plant growth and development, including reproductive processes such as seed development and maturation (Shani et al. 2024). The biosynthesis of GAs occurs via the terpenoid pathway (Fig. 4A). Through a series of enzymatic steps, geranylgeranyl diphosphate (GGDP) is converted to GA_12_, which is a direct precursor to all bioactive GAs in plants (Yamaguchi et al. 1998; Helliwell et al. 1998, 1999, 2001b, 2001a; Sun 2008; Sponsel and Hedden 2010). The conversion of GA_12_ to GA_4_ and GA_7_, involves a series of hydroxylation reactions, which are catalyzed by various 2-oxoglutarate-dependent dioxygenases (2ODDs), including GA 20-oxidases (GA20ox) (Phillips et al. 1995) and GA 3-oxidases (GA3ox) (Hu et al. 2008). GA20ox enzymes catalyze sequential oxidation steps that remove carbon-20 from GA_12_ to produce GA_9_ and GA_20_ (Sun 2008). Following the actions of GA20ox, GA3ox enzymes hydroxylate GA_9_ and GA_20_ at the C-3 position, producing the bioactive GA_4_ and GA_1_, respectively (Hedden et al. 2001).

The first step in GA signaling involves the perception of the bioactive hormone by its receptor, GA INSENSITIVE DWARF1 (GID1) (Fig. 4B). GID1 is a soluble intracellular receptor that undergoes a conformational change upon GA binding, enabling it to interact with downstream signaling components (Ueguchi-Tanaka and Matsuoka 2010; Livne and Weiss 2014). Once GA binds to GID1, the GID1-GA complex interacts with DELLA proteins (Fig. 4B). DELLAs are a family of nuclear-localized repressors of GA responses (Ueguchi-Tanaka et al. 2007; Murase et al. 2008). Their name derives from a conserved DELLA motif present in all family members (Vera-Sirera et al. 2016). The interaction between the GID1-GA complex and DELLA proteins marks the latter for degradation via the 26S proteasome pathway. This process involves the ubiquitination of DELLAs by SCF (SKP1, Cullin, F-box containing complex) (McGinnis et al. 2003; Dill et al. 2004; Fu et al. 2004). The degradation of DELLAs effectively removes the repression on GA signaling, allowing for the activity of TFs and other signaling molecules that promote GA responses (Fig. 4B).

### Regulation of seed development by GA

During seed development, GAs have been mostly implicated in seed coat formation, as we discuss below. However, there are indications that GA related genes are expressed in other seed compartments. Studies into GA biosynthesis in Arabidopsis siliques have highlighted distinct expression patterns of *GA3ox3:GUS* and *GA3ox4:GUS* (Hu et al. 2008). *GA3ox3:GUS* expression was notably confined to developing embryos, particularly during the heart and torpedo stages, with strong activity observed on the interface between the cotyledons and the hypocotyl (Hu et al. 2008). Conversely, *GA3ox4:GUS* activity was predominantly detected in the endosperm of early developing seeds, becoming localized at the chalazal endosperm by the heart stage (Hu et al. 2008). Similarly, the spatial and temporal expression of *GA20oxs* in ovules and developing seeds was also investigated. While *GA20ox1:GUS* expression was restricted to pollen and pollen tubes, and in the proximal site of the embryo sac post-fertilization, *GA20ox2:GUS* exhibited expression in both pollen tubes and seeds, with a shift in spatial expression from the embryo sac to the chalazal pole of the developing seed (Gallego-Giraldo et al. 2014).

In line with those findings, GAs have been proposed to have roles during seed formation, as fertilization induces the expression of several GA biosynthesis genes in the ovules (Dorcey et al. 2009). This also coincides with the removal of the DELLA repressor GFP:RGA from the ovules, indicating post-fertilization activation of GA signalling (Dorcey et al. 2009). Other genes encoding DELLA proteins, like *GAI* and *RGL2* are also expressed in unfertilized ovules (Gallego-Giraldo et al. 2014), but whether the respective proteins are also removed following fertilization has not been assessed. The observation that RGA is specifically expressed in the integuments and removed after fertilization, linked activation of GA signalling to seed coat development (Fig. 4C) (Figueiredo et al. 2016). This was confirmed via the exogenous application of GA3 to ovules and by the ectopic expression of the GA biosynthesis gene *GA3ox1* in the integuments: in both cases, seed coat formation initiates, even without fertilization (Figueiredo et al. 2016). This phenomenon of asexual seed coat formation is also observed in mutants for sporophytically-acting PRC2s and, importantly, this phenotype coincides with the ectopic expression of GA-related genes in those mutants (Roszak and Köhler 2011; Figueiredo et al. 2016). This places GA signalling during seed coat formation downstream of PRC2 activity and, therefore, downstream of auxin. As we discussed above, post-fertilization auxin production leads to PRC2 removal. Which then, in turn, leads to expression of GA biosynthesis genes. Indeed this is in line with observations that auxin applications to ovules trigger GA-related processes, but that GA3 applications do not induce the expression of the auxin reporter *DR5rev* (Dorcey et al. 2009; Figueiredo et al. 2016). These observations coincide with the expression of genes encoding GA receptors (Gallego-Giraldo et al. 2014). Both *GID1A* and *GID1B* are strongly expressed in Arabidopsis ovules (Gallego-Giraldo et al. 2014). Coinciding with this, *gid1* mutants have reduced seed sets, and this seems to be determined by a maternal effect (Gallego-Giraldo et al. 2014). However, whether this effect is due to ovule defects or to post-fertilization loss of GA signalling, is yet to be determined.

There is further evidence that GAs control the development of the sporophytic tissues of the seeds, thereby controlling seed size. In a recent study, reciprocal crosses between WT plants and gain- or loss-of-function DELLA mutants, like *gai-1* and *gaiT6*, respectively, indicated that DELLAs can have a positive effect on seed size (Fig. 4C) (Gomez et al. 2023). Notably, DELLA proteins were found to regulate seed size by promoting growth in ovules and in developing seeds, a process mediated by increased cell proliferation within the integuments (Gomez et al. 2023). Specifically, the gain-of-function *gai-1* mutant exhibited significantly more cells in both outer and inner integuments cell layers compared to the WT, indicative of GAI-mediated regulation of seed coat size through enhanced cell proliferation in maternal integuments (Gomez et al. 2023). These results seem contradictory to what was described for RGA, which is removed from the integuments after fertilization, supposedly relieving the repression of GA-target genes (Dorcey et al. 2009; Figueiredo et al. 2016). However, it is possible that this effect on seed growth could be due to pre-fertilization effects of DELLAs, which lead to more proliferative integuments. Alternatively, it is possible that DELLAs have an initial role in repressing seed coat formation in early stages of seed development, but a positive one in later stages. Interestingly, the gene encoding the growth regulator AINTEGUMENTA (ANT) is a potential target of GAI, as determined by ChIP-Seq analyses (Barro-Trastoy et al. 2022; Gomez et al. 2023). Overexpression of *ANT* in unfertilized ovules leads to more proliferative integuments and, thus, larger seeds (Mizukami and Fischer 2000), supporting the hypothesis of a pre-fertilization DELLA-mediated effect on seed size.

In comparison to their role in the development of sporopyhytic tissues, the effect of GAs in the development of the zygotic products, embryo and endosperm, is not as well established. In pea, loss of zygotic GA biosynthesis coincides with seed abortion phenotypes (Swain et al. 1997), and similar observations were reported in tomato (Groot et al. 1987). Indeed, genes encoding DELLA proteins are expressed in the endosperms of some monocots, like rice and maize (Florez-Rueda et al. 2023), and a *GA20OX* gene is imprinted in the endosperm of water lilies (Povilus et al. 2024). GAs were also reported to be produced in the endosperm of bean and chayote seeds (Nagl 1971; Ceccarelli and Lorenzi 1983). However, to our knowledge, functional studies aimed at understanding the role of GAs during early endosperm development are still lacking.

## Cytokinins regulate seed size and endosperm proliferation

### Cytokinin biosynthesis and signalling

**Fig. 5:**
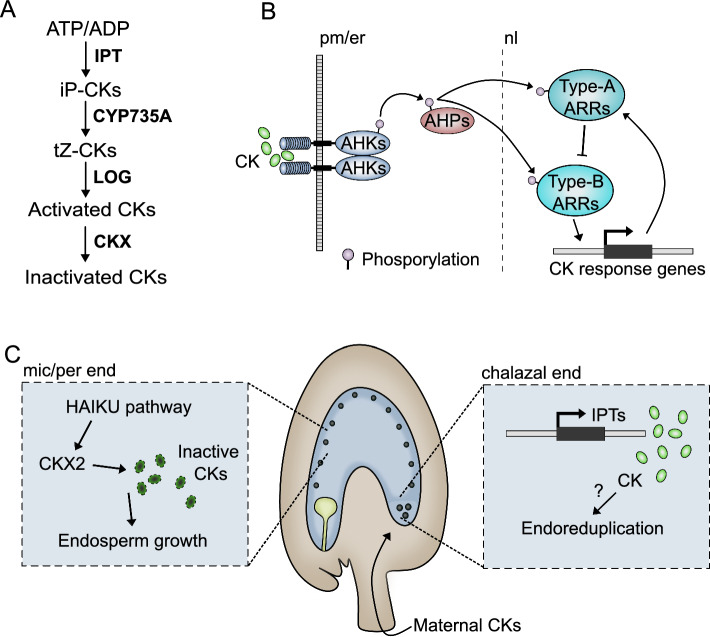
CKs regulate the development of different endosperm regions. **A** CK biosynthesis, activation, and inactivation pathway. **B** CKs are perceived by AHK receptors in the plasma membrane (pm) or at the endoplasmic reticulum (er), and phosphorylation cascades result in the activation of Type-B ARRs, which activate CK-response genes. In turn, Type-A ARRs are also activated and serve as negative regulators of CK-responses, working in a negative feedback manner. **C** In Arabidopsis, CKs are negative regulators of micropylar and peripheral coenocytic endosperm formation (mic/per end), and CK inactivation by CKX2 is under the control of the HAIKU pathway. However, CK biosynthesis genes are strongly expressed in the chalazal endosperm, and high CK levels may lead to the endoreduplication observed in this endosperm region. CKs are also likely provided maternally, via the vasculature

Cytokinins are phytohormones that are intrinsically linked to the promotion of cell division, although they are known to fulfill other roles during plant development (Kieber and Schaller 2018). The primary pathway for cytokinin (CK) biosynthesis in Arabidopsis involves the conversion of adenine nucleotides to CKs (Fig. 5A). This process is catalyzed by the enzyme isopentenyl transferase (IPT), which adds an isopentenyl group to adenosine-5'-triphosphate (ATP) or adenosine-5'-monophosphate (AMP), leading to the formation of isopentenyladenine (iP)-type cytokinins. There are nine IPT genes in the Arabidopsis genome, each playing a role in different tissues or developmental stages (Terceros et al. 2020). After the initial step, the iP-type CKs may be further modified through hydroxylation, side-chain cleavage, conjugation, or oxidation, leading to the formation of various active and inactive forms of CKs. The cytochrome P450 monooxygenase (CYP735A) enzymes are responsible for hydroxylating iP-type CKs to produce trans-zeatin (tZ)-type CKs, which are among the most active forms of these hormones in Arabidopsis (Takei et al. 2004).

Once synthesized, CKs in their inactive forms, such as CK ribosides, must be activated. This activation involves the removal of a ribose group, a reaction catalyzed by CK-specific phosphoribohydrolases, known as LONELY GUY (LOG) enzymes (Fig. 5A). LOG enzymes directly convert inactive CK nucleotides to their free base forms, which are biologically active (Kurakawa et al. 2007). The degradation of CKs is also crucial for maintaining their optimal levels within the plant. This process is primarily carried out by CKs oxidase/dehydrogenase (CKX) enzymes, which catalyze the irreversible breakdown of CKs (Fig. 5A) (Bilyeu et al. 2001; Werner et al. 2003). CKX enzymes remove the isoprenoid side chain from CKs, effectively inactivating them (Mok and Mok 2001). The Arabidopsis genome contains seven *CKX* genes, indicating a complex regulation of CK degradation (Terceros et al. 2020).

In Arabidopsis, CKs are perceived through a specific signal transduction pathway initiated by histidine kinase receptors located on the plasma membrane and at the membrane of the endoplasmic reticulum (Wulfetange et al. 2011; Caesar et al. 2011; Romanov et al. 2018; Antoniadi et al. 2020). The key receptors involved in this process are the Arabidopsis Histidine Kinases 2, 3 and 4 (AHK2-4) (Fig. 5B). These receptors bind CKs and undergo autophosphorylation (Mähönen et al. 2006). The phosphate group is then transferred to an aspartate residue, either within the receptor itself or to an intermediate histidine phosphotransfer protein (AHP) (Inoue et al. 2001; Yamada et al. 2001). The AHPs relay the phosphate group to response regulators (RRs) in the nucleus (Hwang and Sheen 2001). There are two main types of RRs involved in CK signaling: Type-A RRs, which are primarily involved in negative feedback regulation of the CK signal, and Type-B RRs, which act as transcription factors to activate the expression of CK-responsive genes (Fig. 5B) (Hwang et al. 2012; Kieber and Schaller 2018).

### The roles of cytokinins during seed development

CKs play a crucial role in seed development, exerting influence over both seed number and size (Jameson and Song 2016). Manipulation of CK levels often results in changes in organ size, including of whole meristems (Bartrina et al. 2011), and the same is true for seeds. For instance, elevated CK levels, achieved through the constitutive overexpression of CK biosynthetic *LOG* genes, have been associated with the production of larger seeds compared to the WT in Arabidopsis (Kuroha et al. 2009). This effect was maternal, as the seed size increase was only observed when the transgenic *CaMV35S::LOG4* plants were used as mothers (Kuroha et al. 2009). However, there are conflicting reports on the effect of CKs on Arabidopsis seed size, which has been recently reviewed (Terceros et al. 2020). For example, CK-insensitive mutants exhibit enlarged seed phenotypes (Riefler et al. 2005; Hutchison et al. 2006; Argyros et al. 2008), which seems contradictory to the effect of *LOG4* overexpression (Kuroha et al. 2009). The same is true for lines over-expressing the CK degrading enzyme CKX1 in the seed sporophytic tissues (Werner et al. 2003). However, whether these effects are due to pre- or post-fertilization effects of CKs in the sporophyte, remains to be determined. It is also possible that some of these effects are due to the fertility defects of CK mutants, because seed size and seed number are often inversely correlated.

CKs have also been shown to be involved in endosperm development (Fig. 5C). Genes encoding components of the CK perception and signalling machinery are strongly expressed in the early Arabidopsis endosperm (Day et al. 2008). Interestingly, *IPT* genes, involved in CK biosynthesis, are strongly expressed in the chalazal endosperm cyst (Miyawaki et al. 2004; Belmonte et al. 2013). This region of the endosperm is quite particular, in that it does not undergo cellularization, but seems to remain as a coenocyte (Nguyen et al. 2000). The nuclei in the chalazal cyst are larger and of higher ploidy than those in the peripheral or the micropylar endosperm regions, and this seems to be due to endoreduplication and nuclei fusion (Baroux et al. 2004). It is thus possible that these characteristics of the chalazal cyst may be linked to a sustained CK biosynthesis and activity, but to our knowledge this has not been assessed. Unlike what happens in the chalazal endosperm, CK levels decrease in other endosperm regions during coenocytic development. For instance, the CK degrading enzyme CKX2 is expressed in the micropylar end of the endosperm (Li et al. 2013). Interestingly, *CKX2* expression is almost abolished in mutants of the IKU pathway, which regulates coenocytic endosperm size (Li et al. 2013). And, consequently, *iku2* mutant endosperms have increased CK activity (Li et al. 2013). This ectopic activity is likely linked to the small size of *iku* seeds, because overexpression of *CKX2* in a *iku2* background partially rescues that phenotype (Li et al. 2013). This suggests that CKs have a negative effect on coenocytic endosperm growth in Arabidopsis (Fig. 5C). However, in rice endosperms, CK levels were shown to correlate with the cell division rate and, thus, the size of the endosperm (Yang 2002). And in wheat, early stages of seed development show a spike in CK abundance, which correlates with the proliferative phase of the endosperm (Jameson et al. 1982). In line with this, exogenous application of the CK zeatin leads to both increased endosperm cell number and to larger seeds in rice (Yang 2002). Therefore, this spike in CKs may coincide with the establishment of the endosperm as a sink tissue, which has been linked to their interplay with cell wall invertases (Fig. 5C) (Rijavec et al. 2009; Jameson and Song 2016). One interesting point is that a significant percentage of the total CKs in developing seeds are thought to be translocated by xylem and phloem and, therefore, have a maternal origin (Emery et al. 2000). Indeed, exogenous supply of CK to roots leads to increased CK levels in the endosperm (Yang 2002). Because different seeds within a fruit may have different fathers but the same mother, there is a conflict for nutrient allocation between the parents (Haig and Westoby 1989). Thus, it is in the mother’s best interest to evenly allocate nutrients to all the progeny. It is tempting to hypothesize that maternally derived CKs, which control the endosperm sink strength, may be a strategy for maternal control of nutrient allocation to each seed. However, it remains to be explained why CKs seem to be negative regulators of endosperm growth in some species, and to promote it in others. Potentially, as is the case with BRs (Lima et al. 2024), it could be that CKs act in a dose-dependent manner, where too low or too high CK levels are detrimental for endosperm proliferation. It is also interesting to note that, in maize endosperm, there is a sharp increase in CK levels in early stages of seed development (Lur and Setter 1993). This is followed by a sharp decrease in CK levels after 10 days after pollination (DAP), which coincides with the accumulation of IAA (Lur and Setter 1993). Thus, in early stages of maize seed development there is a high CK/Auxin ratio, which is inverted at later stages. Whether the ratio between these two hormones is crucial for proper endosperm development, remains to be tested.

In addition to their role in shaping early stages of endosperm growth, CKs have been implicated in setting the time of endosperm cellularization. In wheat, mutations in *TaMADS-GS*, which encodes a MADS-box TF, result in slightly delayed endosperm cellularization, which correlates with the upregulation of *CKX* genes, involved in CK degradation (Zhang et al. 2024). Interestingly, *CKX* genes seem to be PRC2 targets, and loss of TaMADS-GS leads to H3K27me3 hypomethylation in these loci, which is likely the reason for their upregulation (Zhang et al. 2024). However, whether CKs are indeed a factor impacting on endosperm cellularization in several species, it remains to be tested.

## Other hormones with known roles in early seed development

There are other hormones which have been implicated in regulating the early stages of seed development. For instance, environmental stresses trigger changes in the bioaccumulation of different hormones in maize kernels (Jones and Setter 2015). However, relatively little is known about the actual roles of some of these hormones in shaping seed development. In this section we briefly mention the roles of ethylene and abscisic acid (ABA) during early stages of seed formation. Both hormones are implicated in later stages of seed development (Young et al. 1997; Linkies et al. 2010), but those stages are not covered in this review.

### Ethylene production as a trigger for synergid and nucellus degeneration

Polygonum-type ovules contain two synergid cells, which secrete pollen tube attractants (Higashiyama et al. 2001). The pollen tube enters through one of the synergids, disrupting it, but one synergid cell remains. This cell needs to be degraded, to avoid that additional pollen tubes enter the ovule, and this degradation is triggered by the fertilization event (Beale et al. 2012; Kasahara et al. 2012). Two TFs involved in ethylene responses, ETHYLENE INSENSITIVE 3 (EIN3) and EIN-LIKE 1 (EIL1), were shown to be necessary for degradation of the persistent synergid, because ovules mutant for *ein3 ein1* allow the entrance of supernumerary pollen tubes (Völz et al. 2013). Moreover, microinjections of the ethylene precursor ACC into the female gametophyte leads to synergid degeneration, linking the production of this hormone to cessation of pollen tube attraction (Völz et al. 2013).

In addition to its role in driving synergid degradation, endosperm-derived ethylene has also been implicated in removal of the nucellar cells (Lombardi et al. 2012). The nucellar tissue of the ovule is mostly absent in the seeds of many angiosperms through a process of programmed cell death (PCD) (Domínguez et al. 2001). In Arabidopsis, this process has been shown to be mediated by the formation of the endosperm (Xu et al. 2016). Interestingly, in chayote seeds, endosperm-derived ethylene was proposed to trigger nucellus degeneration, as application of inhibitors of ethylene biosynthesis inhibits the PCD process (Lombardi et al. 2012). Interestingly, a persistent nucellus is also observed in the ovules of the *BAK1* gain-of-function mutant *elg* (Lima et al. 2024). Because BAK1 has been shown to participate in ethylene responses (Kørner et al. 2013), it is possible that BRs and ethylene also interact during nucellus degeneration. Together, these observations place ethylene as a post-fertilization mechanism that non-cell autonomously dictates the fate of accessory cells and tissues, which are no longer required for seed formation.

Finally, factors involved in ethylene signalling have been linked to seed growth and to endosperm cellularization (Ando et al. 2023). Mutations in the gene encoding the membrane protein EIN2, involved in ethylene signalling, lead to larger seeds and possibly to delayed endosperm cellularization (Ando et al. 2023). However, these *ein2* phenotypes seem to be independent of ethylene, as independent mutations in other ethylene-related effectors do not lead to similar phenotypes.

### Abscisic acid regulates the timing of endosperm cellularization

ABA has very well-established roles in seed maturation, dormancy, and germination (Rodríguez-Gacio et al. 2009; Sano and Marion-Poll 2021; Ali et al. 2022). However, its roles in early seed development have been comparatively less explored. Nevertheless, ABA has been linked to the growth of the coenocytic endosperm and to its cellularization. Arabidopsis mutants for *ABSCISIC ACID DEFICIENT 2* (*ABA2*) produce larger seeds, and this phenotype correlates with the upregulation of *SHORT HYPOCOTYL UNDER BLUE 1* (*SHB1*), which encodes a positive regulator of the IKU pathway (Zhou et al. 2009; Cheng et al. 2014). Conversely, overexpression of *ABA2* leads to the downregulation of *SHB1* and of other members of the IKU pathway (Cheng et al. 2014). Seeds mutant for *aba2* also show a delay in endosperm cellularization, which is consistent with the upregulation of the IKU pathway (Cheng et al. 2014). Consistent with a role of ABA in cellularization, the failure of this important developmental transition has been linked to an osmotic stress response in embryos (Xu et al. 2023). Failure of endosperm cellularization is often observed in seeds from crosses of diploid mothers with a father of higher ploidy (Scott et al. 1998). Consistent with previous reports (Cheng et al. 2014), this cellularization phenotype can be rescued by inhibition of ABA catabolism, and exacerbated by mutations that abolish its biosynthesis (Xu et al. 2023). The role of ABA in endosperm formation seems to be conserved, as barley mutants with low ABA levels also show seed filling defects and abnormal endosperm cellularization phenotypes (Sreenivasulu et al. 2010). Interestingly, similar to CKs, ABA is exported from the leaves into maize and rice seeds (Ober and Setter 1990; Qin et al. 2021). Thus, because of the role of ABA in setting the timing of cellularization, it is possible that maternally derived ABA can be a vegetative signal that contributes to determining resource allocation to developing seeds.

## Conclusions/perspectives

The endosperm and the seed coat are accessory structures whose main function is to nourish and to protect the embryo, while not contributing themselves to this new generation. Moreover, these two structures also contribute to determining final seed size, which is an important physiological and agricultural trait. Thus, the coordinated and tuned development of the endosperm and of the seed coat is of utmost importance for successful seed development. This explains why their development is under such tight genetic regulation, and why bi-directional communication between the two is critical to establish a healthy seed. Importantly, phytohormones dictate many aspects of endosperm and seed coat development, including how they communicate with one another. As we point out throughout this manuscript, a tightly controlled hormonal homeostasis and intricate crosstalk between different hormones are critical factors in shaping seed development. This balance is crucial in determining seed survival, and its manipulation is thus a powerful tool for future crop improvement.
